# Effect of Sodium Acetate on High-Temperature Gelation Characteristics of Sodium-Modified Calcium-Based Bentonite Water-Based Drilling Fluids

**DOI:** 10.3390/gels12030238

**Published:** 2026-03-13

**Authors:** Rui Liu, Yu Zhao, Huan Wang, Wenjun Long, Junge Zhu, Fengshan Zhou

**Affiliations:** 1 Beijing Key Laboratory of Materials Utilization of Nonmetallic Minerals and Solid Wastes, National Laboratory of Mineral Materials, School of Materials Science and Technology, China University of Geosciences (Beijing), No. 29 Xueyuan Road, Haidian District, Beijing 100083, China; 2003240034@email.cugb.edu.cn (R.L.);; 2 China National Petroleum Corporation (CNPC) Dushanzi Petrochemical Branch, No. 6 Beijing Road, Dushanzi District, Karamay 833699, China; 3 Xinjiang Geological Bureau Mineral Experiment Research Center, No. 388 Kelamayi West Road, Shayibake District, Urumqi 830000, China; 4 Key Laboratory of Carrying Capacity Assessment for Resource and Environment, Ministry of Natural Resources of the People’s Republic of China, School of Economics and Management, China University of Geosciences (Beijing), No. 29 Xueyuan Road, Haidian District, Beijing 100083, China

**Keywords:** sodium acetate, gelation, high-temperature resistance, water-based drilling fluid, bentonite

## Abstract

As global oil and gas exploration extends to deep and ultra-deep wells, high bottom-hole temperature is prone to deteriorating the gelation and rheological properties of water-based drilling fluids, which manifests as undesirable thickening or thinning at elevated temperatures. Therefore, the development of high-temperature resistant and stable drilling fluids is crucial for ensuring safe and efficient drilling operations, and the enhancement of high-temperature performance is typically achieved by adding drilling fluid treatment agents. The main objective of this study is to apply sodium acetate (SA) to drilling fluid systems, developing an economical and efficient non-polymer treatment agent with dual functions as a composite sodium-modifier and a rheological regulator. By-product sodium acetate (TRSA) is adopted to provide better cost-effectiveness while maintaining equivalent performance, and its universality across seven types of bentonites is verified. Three grades of sodium acetate were added to the bentonites as either composite sodium-modifiers or rheological regulators. After high-temperature aging, rheological parameters, including mud density, plastic viscosity (PV), yield point (YP), and gel strength, were measured in accordance with standard API methods. The results indicate that adding 2 wt.% TRSA to drilling fluid and subjecting it to hot rolling at 180 °C for 16 h keeps the viscosity at a high shear rate (1022 s^−1^) nearly unchanged (from 36 mPa·s to 37.5 mPa·s), while increasing the viscosity at a low shear rate (5.11 s^−1^) from 250 mPa·s to 1400 mPa·s, thereby effectively improving the shear thinning effect of the sodium-modified calcium-based bentonite water-based drilling fluid. Although TRSA increases the filtration loss from 21.8 mL to 30 mL, this can be reduced to 20–25 mL by co-extrusion sodium modification with sodium carbonate or by adding additional TRSA to sodium-modified bentonite. This study provides a novel perspective for significantly improving the gelation characteristics and rheological properties of bentonite suspensions at high temperatures through a special inorganic substance, while realizing resource reuse and cost reduction.

## 1. Introduction

The development potential of global oil and gas resources is enormous, and the position of oil and gas resources in the energy structure is still difficult to be quickly replaced in the short term [1]. With the continuous development of science and technology, oil exploration technology is also constantly improving. Oil and gas resources have been discovered in many unexplored areas, such as deep strata, deep water and shale [2]. China Petroleum conducted an analysis of global oil and gas resources in 2020. The recoverable resources of conventional oil and gas in the world are 6415.7 × 10^8^ t, and the total recoverable resources of unconventional oil and gas in the world are 6352.3 × 10^8^ t [3]. With the gradual improvement and development of science and technology, the exploration, development, and utilization of oil and gas resources will gradually increase [4,5]; research into high-temperature resistant treatment agents has become increasingly crucial to safeguarding the efficiency and safety of deep drilling operations.

Due to the continuous increase in global oil and gas consumption [6], the proportion of deep and ultra-deep oil wells has steadily increased in recent years [7]. Usually, deeper wells are accompanied by higher bottom-hole temperatures (>148 °C) [8], which greatly increases the cost and risk of drilling [9]. This requires drilling fluids with better performance to meet the needs of drilling operations at high temperatures. Drilling fluid is usually composed of bentonite, various treatment agents and weighting agents [10,11]. The key to designing drilling fluid systems is to control the rheology and filtration properties of the drilling fluid [12]. Therefore, the development of high-temperature resistant drilling fluids, which can maintain stable rheology and filtration control even under extreme downhole temperatures, has become a critical focus to address the challenges of deep drilling and ensure operational efficiency and safety. High temperature will lead to the dealumination and reduction of hydroxyl groups on the surface of clay, thus reducing the negative conductivity and hydrophilicity of clay. This increases the size of clay particles in the drilling fluid, resulting in thicker and looser filter cakes. Therefore, the rheological and filtration properties of drilling fluid either decrease or are destroyed [13]. Drilling fluid treatment agents would be damaged by degradation, crosslinking and desorption at high temperatures, resulting in a serious decline in drilling fluid performance, thus affecting the efficiency of drilling operations [14]. In order to maintain good rheological properties and filtration loss of drilling fluid at high temperatures, a high-temperature treatment agent is mainly added to the drilling fluid to stabilize the performance of the drilling fluid. Current high-temperature-resistant treatment agent systems are mainly classified by their core materials into organic polymer-based, inorganic, biological derivative-based, and composite systems. Domestically and internationally, research has long focused on enhancing the high-temperature stability of polymer-based treatment agents, including anionic polymers [15], cationic polymers [16], amphoteric ion polymers [17], and nonionic polymers [18]. However, polymer-based treatment agents inherently suffer from limitations such as vulnerability to thermal degradation and unbalanced rheological regulation at high temperatures. Current studies have shown that materials such as hydrophobic associative network-structured polymer ASML [19], zwitterionic quaternary copolymer DADN [20], psyllium husk [21], modified xanthan gum derivative XG-g-AAA [22], and star-shaped AM-AMPS copolymer [S-poly(AM-co-AMPS)] [23] can enhance the high-temperature resistance of drilling fluids through mechanisms like adsorption and dispersion, network structure formation, and hydration enhancement, while optimizing their viscosity, colloidal stability, filtration control, and shear thinning properties. However, adding polymers to bentonite mud to enhance the viscosity and shear stress of drilling fluid can mostly maintain the apparent viscosity at a high shear rate (1022 s^−1^) after high-temperature aging, but it is difficult to maintain the viscosity at a low shear rate (5.11 s^−1^). In other words, the maintenance of the gel strength required for cuttings suspension after high-temperature aging necessitates the addition of polymer, which induces excessively high viscosity at high shear rates, thereby compromising drilling speed and efficiency. In contrast, inorganic high-temperature stabilizers function through ionic regulation and crystal structure stability, avoiding such issues [24]. For instance, synthetic hectorite can maintain a viscosity of over 20 mPa·s at temperatures above 200 °C [25], but it has shortcomings, including limited enhancement of gel strength at low shear rates and high synthesis costs. Sodium-based inorganic salts are widely used as sodium-modifying agents but lack independent rheological regulation capabilities, requiring combination with other additives to achieve comprehensive performance.

**Figure 1:**
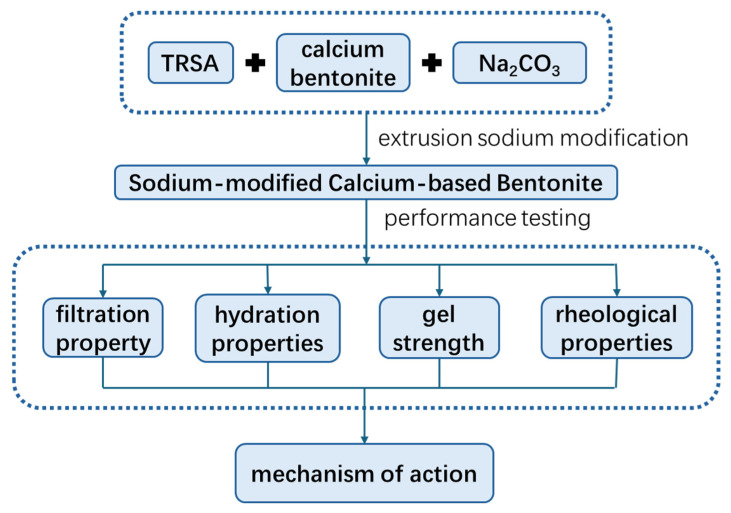
Technical route flow chart of sodium acetate-modified bentonite drilling fluid.

In this study, the rheological properties of drilling fluid before and after high-temperature aging, the hydration properties of bentonite, and the stability of drilling fluid colloids were analyzed [26]. The effects of different grades of sodium acetate as a part of the sodium agent and as a rheological modifier on bentonite and drilling fluid systems were explored. The effect of sodium acetate on the maintenance of high-temperature stability of drilling fluids in the system was analyzed through multiple sets of cross-experiments. The utilization of bentonites with different origins and qualities demonstrated that the high-temperature resistance effect of sodium acetate can be applied to various types of bentonites, exhibiting good universality. The specific process can be seen in Figure 1. A new idea was proposed to maintain the high-temperature stability of drilling fluid and improve the gel strength of drilling fluid at high temperatures.

## 2. Results and Discussion

### 2.1. Effect of Sodium Acetate Type on Drilling Fluid Performance

**Figure 2:**
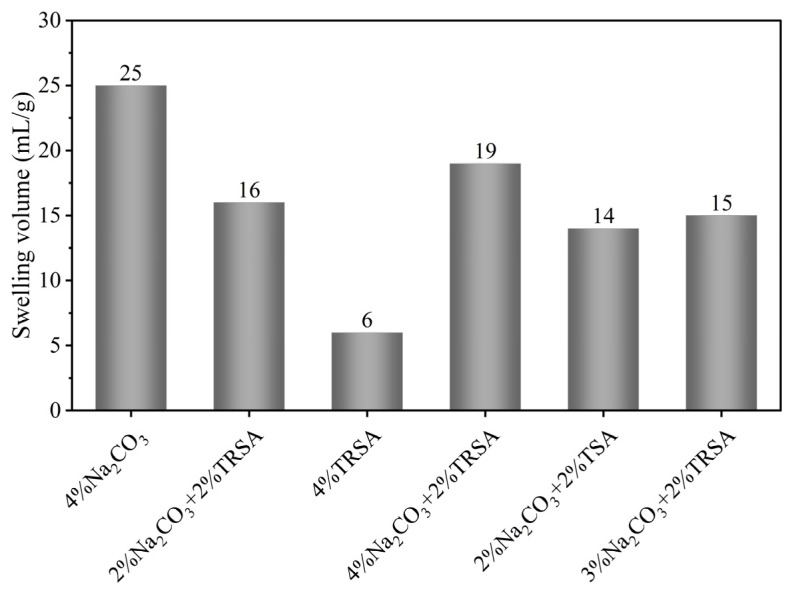
Swelling volume of bentonite sample DG for different sodium agents.

Most of the bentonite in China is calcium-based bentonite, which has poor hydration performance [27,28]. To be applied in the drilling fluid industry, it is necessary to modify it with sodium to become sodium-based bentonite. Bentonite DG is one of the standard soils used in the drilling fluid industry. In the experiment, six different sodium agents were designed using different ratios of sodium carbonate and sodium acetate. The sodium modification of DG was carried out, and its swelling volume and rheological properties were tested after modification. Figure 2 shows the expansion capacity of DG under the action of different sodium agents, which can represent the sodium effect of sodium agents on bentonite to a certain extent. For most bentonites, the optimum dosage of Na_2_CO_3_ is 3–5% [29]. It was observed that, for bentonite DG, the hydration expansion effect of bentonite is best when using traditional 4% Na_2_CO_3_ to sodium-modify it. However, using 2% by-product grade sodium acetate TRSA instead of half Na_2_CO_3_ under the expansion capacity index did not achieve the effect of 4% Na_2_CO_3_ as a sodium agent, because sodium acetate could not provide carbonate that could precipitate Ca^2+^ and the influence of impurity metal ions contained in by-product sodium acetate exerted an adverse influence, resulting in a low swelling volume. When all by-product sodium acetate was used to replace sodium carbonate, as there was not enough carbonate to move the sodium balance to the right, a large amount of Ca^2+^ in the interlayer of calcium-based bentonite was not replaced, resulting in poor sodium effect, and thus, rendering it unusable in drilling fluid. When an additional 2% TRSA was added to 4% Na_2_CO_3_, it provided more sodium ions, which exceeded the optimal amount of sodium ions required for bentonite-sodium modification, and impurities in the by-product sodium acetate, resulting in a certain decrease in swelling volume compared to using 4% Na_2_CO_3_ as a sodium modification agent [30]. However, since enough carbonate could be provided, the effect of sodium modification was better than that of using 2% TRSA to replace sodium carbonate. Compared with the by-product sodium acetate TRSA, the swelling volume of industrial-grade sodium acetate TSA was not significantly different. Under the same conditions using 6% composite sodium agent, the hydration expansion effect of 4% Na_2_CO_3_ + 2% TRSA was better than that of 3% Na_2_CO_3_ + 3% TRSA.

**Figure 3:**
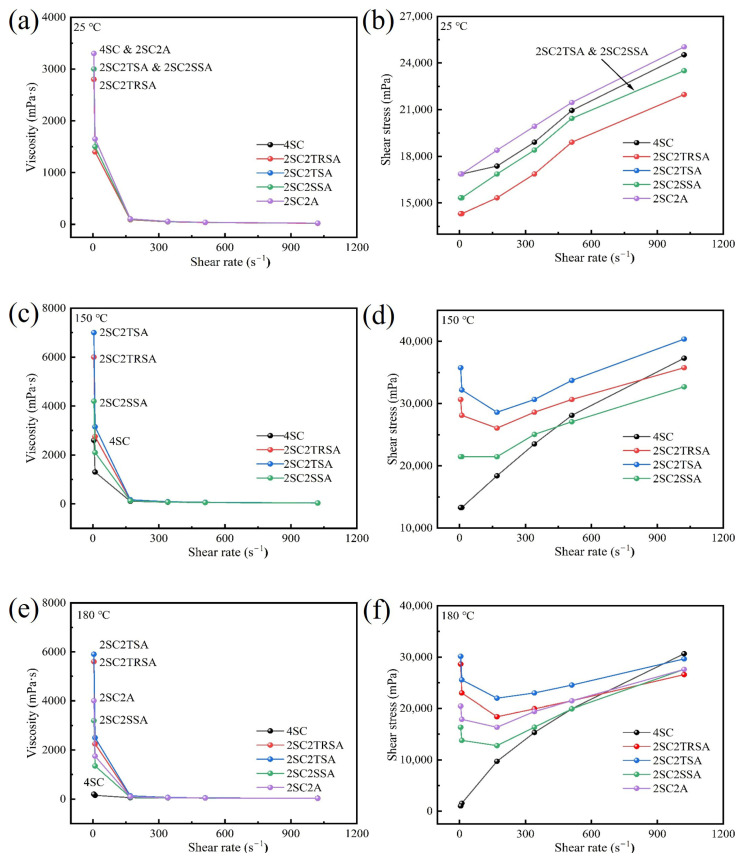
Rheological properties of bentonite DG after aging at room temperature and high temperature using different sodium agents: (**a**) 25 °C viscosity curve; (**b**) 25 °C shear stress curve; (**c**) 150 °C viscosity curve; (**d**) 150 °C shear stress curve; (**e**) 180 °C viscosity curve; (**f**) 180 °C shear stress curve.

Table 1 shows the composition of five sodium agents. Figure 3 shows the rheological properties of drilling fluid prepared by bentonite sample DG using different sodium agents at 25 °C, 150 °C and 180 °C. It can be seen from Figure 3a,b that the rheological properties of the drilling fluid mud obtained by using different sodium agents to sodium DG at 25 °C are roughly the same. The effects of TRSA, TSA, SSA and A were similar. The above experiments proved that whether using by-product sodium acetate or industrial grade sodium acetate and analytically pure sodium acetate, the rheological properties after pulping at room temperature could replace the traditional 4% Na_2_CO_3_ measurement. Figure 3c,d illustrate the data from drilling fluid aging at 150 °C, while Figure 3e,f show the data from drilling fluid aging at 180 °C. From the rheological property diagram of high-temperature aging, it can be seen that the apparent viscosity and shear stress of drilling fluid with 4% Na_2_CO_3_ at low shear rate after high-temperature aging are greatly reduced, which will lead to a reduction in the effect of drilling fluid on suspending cuttings. If the drilling fluid cannot effectively suspend cuttings and weighting materials, they will sink and accumulate at the bottom of the well, causing the drill to repeatedly break the cuttings, thus slowing down the drilling speed. This can even lead to sticking, which would make drilling impossible. At this point, additional suspending agent would be needed to improve the suspension capacity of the drilling fluid, but this would increase the drilling cost. However, drilling fluid prepared with different sodium acetate composite sodium agents can maintain good apparent viscosity and shear stress after high-temperature aging. From the curves shown in Figure 3d,f, it can be seen that the use of sodium acetate composite sodium agents can maintain high shear stress in the drilling fluid at low shear rate and reduce shear stress at high shear rate at high temperatures, which represents an improved shear dilution effect in the drilling fluid. This allows the drilling fluid to better suspend cuttings while reducing bit wear and improving the efficiency of drilling operations. Table 2 shows the dynamic ratio and filtration loss of bentonite DG drilling fluid obtained from using different sodium agents. According to the dynamic speed ratio data, the changes in dynamic speed ratio before and after high-temperature aging are smaller than those of a traditional sodium agent, which also proves the improvement of drilling fluid temperature resistance performance. From the data on filtration loss, it can be seen that the use of the composite sodium agent increases the filtration loss of the bentonite drilling fluid. There is a certain relationship between the filtration loss of the drilling fluid and the hydration performance of bentonite; the hydration performance of the drilling fluid using composite sodium agents was worse than that of the drilling fluid using pure sodium carbonate, which led to an increase in filtration loss. This may be due to the failure of sodium ions in sodium acetate to effectively replace calcium ions between the bentonite layers, resulting in their dissociation in the drilling fluid system. The use of a composite sodium agent and filtrate reducer can improve the problem of increased filtration loss to a certain extent [31].

### 2.2. The Influence of the Sodium Reaction Method of the Composite Sodium Agent on the Drilling Fluid

**Figure 4:**
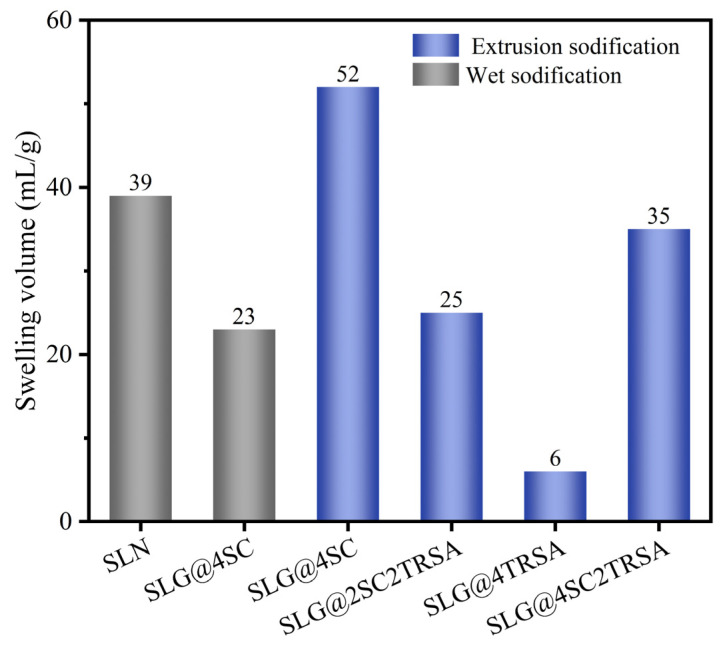
The swelling volumes of bentonite SLN and SLG under different sodium modification methods.

Figure 4 shows the swelling volumes of two kinds of bentonite SLN and SLG from Shengli Oilfield under different sodium methods. SLN is a bentonite used for drilling fluid after SLG has been formulated with various drilling fluid treatment agents, while SLG is a calcium-based bentonite without the addition of treatment agents. It can be seen from the swelling volume data that the extrusion sodium modification had an immediate effect on the hydration expansion performance of bentonite. Extrusion sodium modification has been shown to greatly enhance the hydration capacity of bentonite and improve the slurry ability of bentonite [32]. The swelling volume of SLG (4SC) increased from 23 mL/g to 52 mL/g after extrusion. Extrusion sodium modification made the layered structure of bentonite staggered, so that more Ca^2+^ between the layers was replaced, thus improving the sodium effect [33]. Through the extrusion sodium modification experiment, it can be observed that the effect of different sodium agents on the swelling volume of SLG is consistent with the results obtained by DG. The sodium effect of 4SC was better than that of 2SC2TRSA and 4SC2TRSA. Compared with the results from wet sodium modification, it can be seen that, after the extrusion sodium modification using 2SC2TRSA composite sodium, the swelling volume was higher than that of the 4SC wet sodium. Therefore, for some bentonites with a general wet sodium effect using a composite sodium agent, the hydration performance could be improved by extrusion sodium modification, thus expanding the application range of the composite sodium agent.

**Figure 5:**
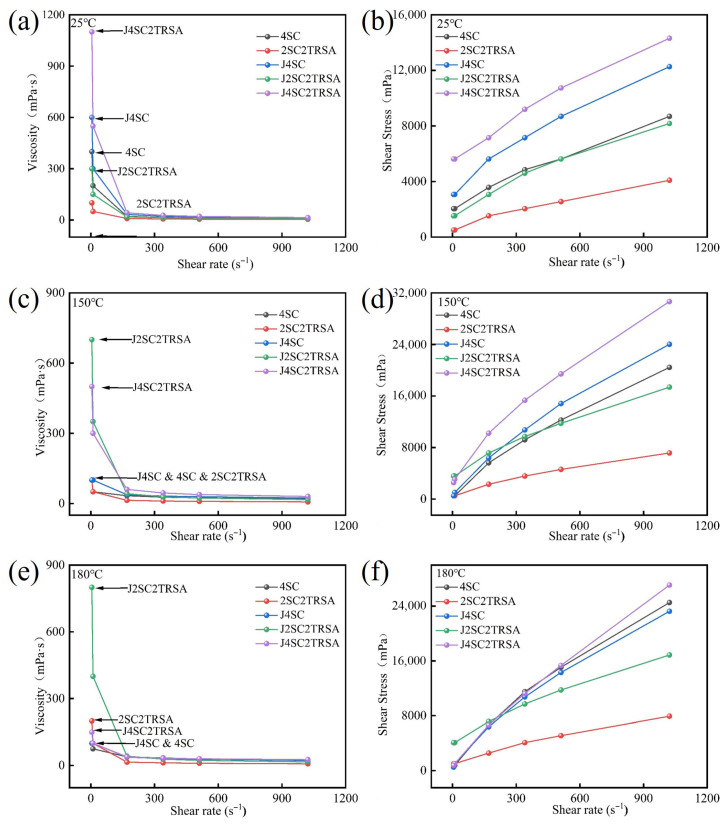
Rheological properties of SLG using composite sodium agent under extrusion sodium modification: (**a**) 25 °C viscosity curve; (**b**) 25 °C shear stress curve; (**c**) 150 °C viscosity curve; (**d**) 150 °C shear stress curve; (**e**) 180 °C viscosity curve; (**f**) 180 °C shear stress curve.

From the red curves shown in Figure 5, it can be seen that for SLG bentonite, the rheological properties of the drilling fluid obtained through wet sodium modification using 2SC2TRSA composite sodium agent were poor. Its viscosity and filtration were not up to the standard of drilling fluid. Figure 5c–f show the rheological data of the drilling fluid after high-temperature aging at 150 °C and 180 °C; 2SC2TRSA and J2SC2TRSA are SLG after wet sodium modification and extrusion sodium modification using composite sodium agent 2SC2TRSA, respectively. From the comparison of the two sets of data, it can be observed that the shear stress of the whole drilling fluid could be improved by using the composite sodium agent after extrusion sodium modification, and the J2SC2TRSA after extrusion sodium modification could better maintain viscosity at low shear rate after high-temperature aging, which is very beneficial to drilling operations. Comparing J4SC and J2SC2TRSA after 25 °C and high-temperature aging, at 25 °C, the viscosity and shear stress of the composite sodium agent at low shear rate and high shear rate were less than those of J4SC. However, after high-temperature aging, the drilling fluid using the composite sodium agent could reduce the viscosity at high shear rate and increase the viscosity at low shear rate, so that the dynamic speed ratio of the drilling fluid (Table 3) had almost no change compared with that at room temperature, demonstrating that temperature resistance was greatly improved. Observing the curve of J4SC2TRSA in the figure, it can be seen that after using 4% Na_2_CO_3_ and adding an additional 2% TRSA, higher apparent viscosity and shear stress could be obtained through extrusion sodium modification.

### 2.3. Effect of Adding Sodium Acetate as Rheological Modifier to Sodium Bentonite on Drilling Fluid Performance

In the configuration of bentonite for drilling fluid, anti-high-temperature filtrate reducers [34,35] and anti-high-temperature shearing enhancers [36] are usually added to make the viscosity and shear stress of drilling fluid reach operating standards. There are various types of bentonites, and the rheological properties of bentonite from different origins vary greatly [37]. Some high-temperature-thickened bentonites exhibit excessively high viscosity at high shear rates after high-temperature aging [38], which leads to a decrease in bit drilling speed. Some high-temperature-diluted bentonites have a significant decrease in viscosity after high-temperature aging, and the effectiveness of the drilling fluid suspension of cuttings is also greatly reduced. Such bentonites can affect drilling efficiency at high temperatures. By utilizing the advantage of sodium acetate to reduce viscosity at high shear rates while improving low shear performance, better-performing bentonites for drilling fluids can be prepared, enabling them to maintain excellent rheological properties even after high-temperature aging.

**Figure 6:**
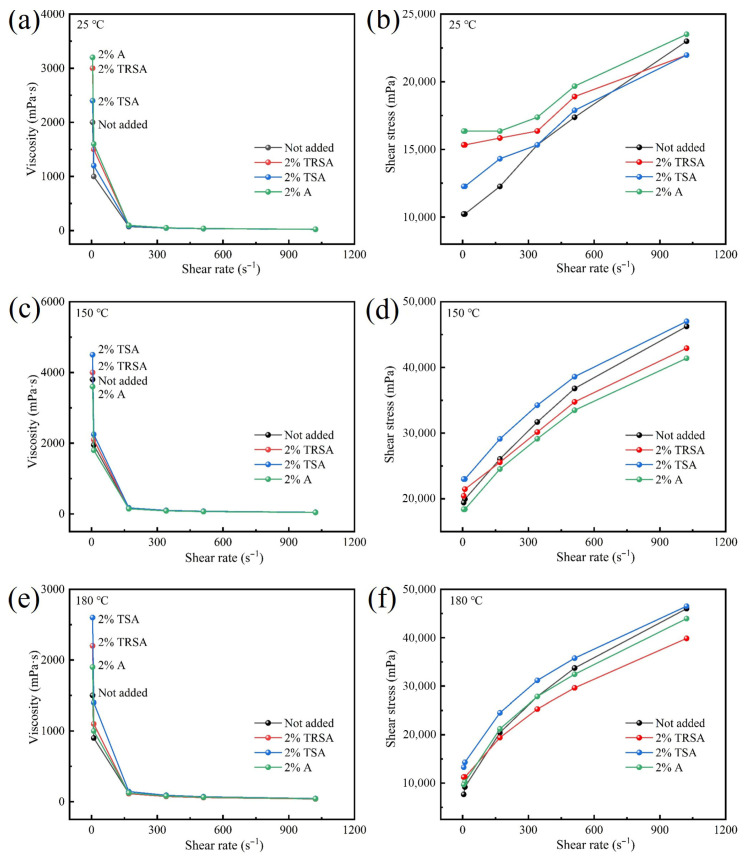
Rheological properties of bentonite JXH after adding different sodium acetate: (**a**) 25 °C viscosity curve; (**b**) 25 °C shear stress curve; (**c**) 150 °C viscosity curve; (**d**) 150 °C shear stress curve; (**e**) 180 °C viscosity curve; (**f**) 180 °C shear stress curve.

JXH is a sodium-modified calcium-based bentonite from Xuanhua, Hebei Province. In an experiment, the rheological properties of drilling fluid prepared by JXH with different grades of sodium acetate (by-product grade, industrial grade and analytical grade) as rheological modifiers were investigated. In Figure 6c, it can be seen that JXH has excellent temperature resistance at 150 °C, and the viscosity at each shear rate can be maintained regardless of whether sodium acetate is used or not. Figure 6e shows the temperature resistance of sodium acetate when the temperature is increased to 180 °C. The low shear rate viscosity of drilling fluid without adding sodium acetate significantly decreases at 180 °C, while several groups of drilling fluid with added sodium acetate can effectively improve the viscosity and shear stress at low shear rates while reducing the viscosity and shear stress at high shear rates, thereby improving the efficiency of drilling operations. Therefore, the use of different types of sodium acetate as rheological regulators is universal for the rheological properties of drilling fluids under high-temperature aging. As shown in the filtration loss data in Table 4, for bentonite that was sodium-treated, the effect of sodium acetate as a rheological modifier on the filtration loss of drilling fluid was smaller than that of sodium acetate as a composite sodium treating agent, with only a 10% increase in filtration loss.

### 2.4. Synergistic Effect of Sodium Acetate as a Rheological Regulator and a Tackifier When Used Together

In the configuration of bentonite for drilling fluid, some types of bentonites may not meet the viscosity and shear strength requirements of drilling fluid performance at room or high temperatures. In order to adjust the viscosity and shear force when designing drilling fluid, it is usually necessary to add tackifier and rheological regulators [39], such as sodium polyacrylate, polyacrylamide [40,41], Xanthan gum [42], CMC [43], MgO, etc.

The rheological data were further analyzed using the Herschel–Bulkley model (Equation (1)), which effectively describes the non-Newtonian behavior of drilling fluids:

τ = τ_y_ + K·γ̇^n^ (1)

where τ is the shear stress (Pa), γ̇ is the shear rate (s^−1^), τ_y_ is the yield stress (Pa), K is the consistency coefficient (Pa·s^n^), and n is the flow index (dimensionless). The parameters were obtained by nonlinear regression fitting of the shear stress–shear rate data measured at six speeds (φ_3_, φ_6_, φ_100_, φ_200_, φ_300_, φ_600_) on the Fann 35 viscometer. All fittings yielded a coefficient of determination (R^2^) greater than 0.99, confirming the model’s adequacy.

Comparing ① and ③ in Table 5, the viscosity and shear stress of drilling fluids were improved by adding sodium polyacrylate. For high-temperature-thickened bentonite such as JGN, the R_600_ value after aging at 150 °C increased from 55.5 to 112, which is almost doubled in increase. The high viscosity at high shear rate makes the drilling operation inefficient. After aging at 180 °C, the values of R_6_ and R_3_ in ③ were only 4 and 2.5, and the decrease in viscosity at low shear rate would reduce the ability of the drilling fluid to suspend cuttings. A comparison between Group ① and Group ② reveals that the addition of sodium acetate effectively reduces viscosity at high shear rates while enhancing viscosity at low shear rates, thereby maintaining high-temperature stability, though the increase in shear force remains modest. Concurrently, sodium acetate also reduces the hydration performance of bentonite, which leads to a decline in filter cake quality and an increase in permeability. Furthermore, by comparing the k-values at different temperatures, it can be concluded that high temperature promotes the aggregation of bentonite particles, resulting in a thicker and looser filter cake structure.

**Figure 7:**
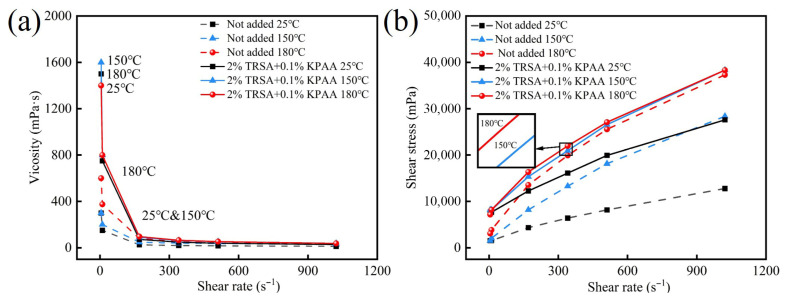
Comparison of rheological properties of JGN before and after using additives: (**a**) Viscosity curve; (**b**) shear stress curve.

Table 6 outlines the increase in flow index (n) values. The data indicate that sodium acetate weakens the shear-thinning behavior of the fluid, meaning the decline in viscosity with increasing shear rate becomes more gradual. In terms of a way to better meet operational requirements for drilling fluid, the data from Groups ① and ④ in Table 5 and the rheological curve in Figure 7 indicate that the combination of sodium acetate and sodium polyacrylate plays a joint role, which can make the rpm value of the drilling fluid at R_600_ remain almost unchanged after aging at 180 °C, while increasing the rpm value at R_6_ from 7.5 to 16. Furthermore, the rheological data of the drilling fluid at 150 °C and 180 °C are almost identical, demonstrating a drilling fluid with good high-temperature stability. Comparing two sets of data, ③ and ④ in Table 6, it can be seen that while ③ substantially increased the yield stress at both room and high temperatures, it exhibited the lowest *n* values (0.62–0.69), indicating pronounced shear-thinning behavior. After aging at 180 °C, its structure was likely compromised, leading to a sharp decline in low-shear-rate performance. In contrast, ④ successfully stabilized the *n* value within a more desirable range of 0.70–0.73 while maintaining a relatively high yield stress. More importantly, all Herschel–Bulkley parameters (τ_y_, K, and n) and conventional parameters (AV, PV, and YP) for ④ were highly consistent between 150 °C and 180 °C. 

### 2.5. Effect of Sodium Acetate Addition on Rheological Properties of Drilling Fluid

**Figure 8:**
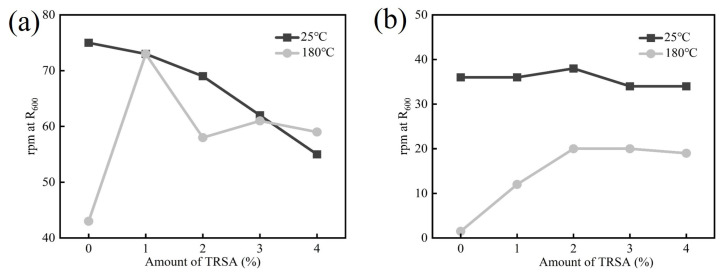
The effect of adding different contents of by-product sodium acetate TRSA to drilling fluid on rpm value: (**a**) The reading of the rotary table at the drilling speed R_600_ of the viscometer at 25 °C and 180 °C; (**b**) the reading of the rotary table at the drilling speed R_3_ of the viscometer at 25 °C and 180 °C.

**Figure 9:**
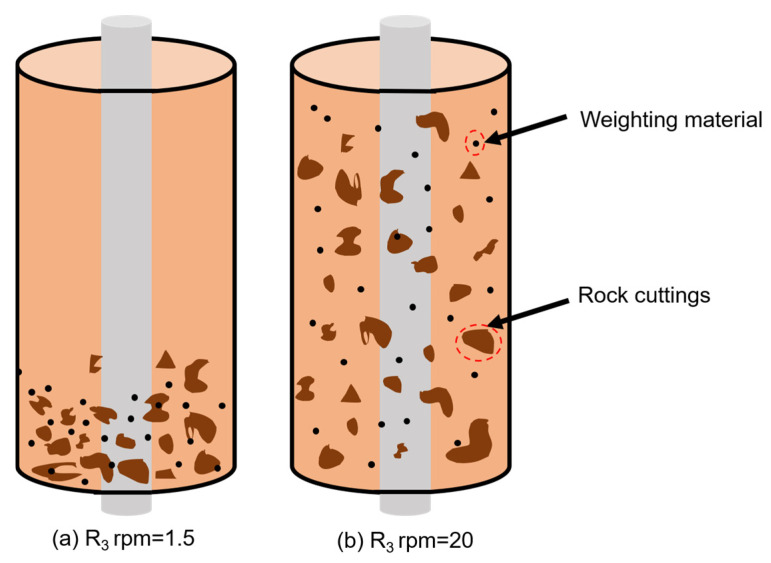
Effects of TRSA on the ability of drilling fluid to suspend cuttings and weighting materials after aging at 180 °C: (**a**) Drilling fluid without sodium acetate; (**b**) drilling fluid with TRSA. Note: Black dots denote weighting material, brown irregular shapes denote rock cuttings, and the orange background represents the drilling fluid.

In order to explore the effect of using bentonite SLN with the addition of by-product sodium acetate TRSA on the rheological properties of drilling fluid, 0% to 4% TRSA was added to the bentonite SLN separately, and the rpm values of the drilling fluid were recorded at different speeds using a viscometer after aging at room temperature and 180 °C. The direct use of viscometer dial readings instead of rheological parameters has been recognized by drilling engineers and technicians. The R_6_ dial reading of drilling fluid is a key factor affecting the transportation of rock cuttings [44]. This reading corresponds to the ultra-low shear rate (5.11 s^−1^) test at 6 rpm, accurately simulating the laminar/plug flow of drilling fluid in the wellbore annulus. Its value directly reflects the low-shear yield stress and structural viscosity of drilling fluid, a core rheological index for cuttings suspension and transportation. A sufficient R_6_ reading indicates a stable weak gel structure in the drilling fluid, which generates viscous drag to counteract cutting gravitational settling, prevents cutting bed formation in deviated/horizontal wells and ensures hole cleaning efficiency. Conversely, an insufficient reading leads to cutting settling, reduced drilling speed and even downhole accidents, underscoring its critical role in regulating cutting transportation. In actual testing, the readings of R_3_ and R_6_ on the viscometer are almost similar or even the same, so the rotary table values of R_3_ and R_6_ have the same effect on directly observing the performance of the drilling fluid. From the data shown in Figure 8a, it can be seen that for SLN bentonite, the rpm value of R_600_ shows a downward trend with the increase in TRSA dosage at room temperature, indicating that adding TRSA can reduce the viscosity and shear stress of drilling fluid at high shear rates at 25 °C. After aging at 180 °C, the temperature resistance of TRSA was demonstrated. From (a), it can be seen that the rpm value of drilling fluid without TRSA decreases sharply after aging. The addition of several sets of drilling fluids with TRSA maintains a certain level of performance: the drilling fluid with an addition amount of 1% produces the same data at 25 °C and 180 °C, and has a good temperature resistance effect. When the addition amount of TRSA exceeds 1%, the rpm value of the drilling fluid at high shear rate decreases after high-temperature aging at 180 °C, playing a role in reducing viscosity at high shear rate. It can be seen from Figure 8b that the addition of TRSA has little effect on the rpm value of drilling fluid R_3_ at 25 °C. At a high temperature of 180 °C, the rpm value of the drilling fluid without TRSA at R_3_ decreases from 36 to 1.5, greatly reducing the cleaning performance of the drilling fluid for rock cuttings. However, the drilling fluid with TRSA can maintain a certain rpm value. When the addition amount exceeds 2%, the rpm value of the drilling fluid at a high temperature of 180 °C can be maintained around 20, as shown in Figure 9, allowing the drilling fluid to effectively suspend rock cuttings at high temperatures, thereby increasing hole cleaning efficiency.

**Figure 10:**
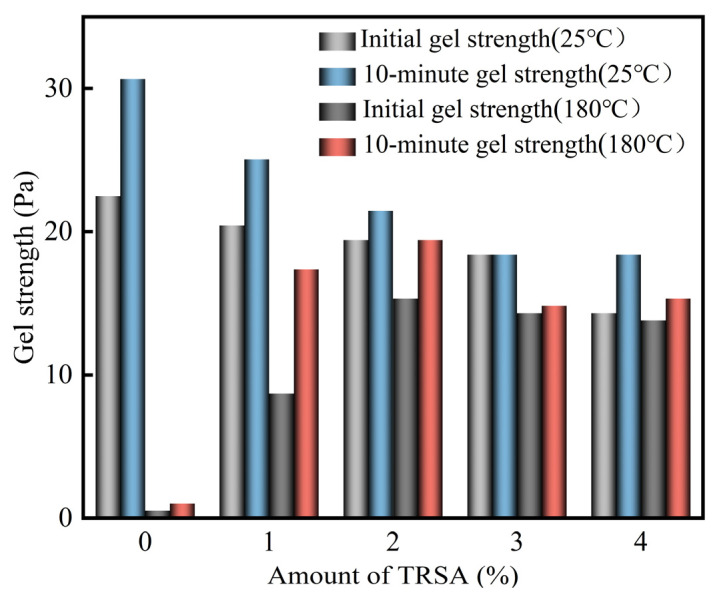
The effect of different contents of sodium acetate TRSA on the initial and 10 min gel strength of drilling fluid (1 lb/100 ft^2^ = 0.4788 Pa).

The gel strength can represent the ability of drilling fluid to suspend cuttings and weighting materials to a certain extent. Weak gel strength makes it so that particles easily settle down, thus representing weak suspension ability [45]. Figure 10 shows the maximum deflection value of R_3_ gear recorded after 10 s of drilling fluid static and the maximum deflection value of R_3_ gear recorded after 10 min of drilling fluid static. From these, the initial and 10 min gel strength of drilling fluid were calculated. It can be seen from the diagram that increasing the amount of TRSA decreases the initial and 10 min gel strength of drilling fluid at 25 °C, but the decrease is not significant. It is known that TRSA has little effect on the gel strength of drilling fluid at 25 °C. However, the difference in initial and 10 min gel strength between drilling fluids without TRSA at room temperature and high temperature is significant, making the performance of such drilling fluids very unstable. When 1% TRSA was added, the drilling fluid could maintain a certain gel strength after high-temperature aging. When the amount of TRSA was increased to 2%, the initial and 10 min gel strength further increased, and the gel strength could be maintained nearly the same as that at 25 °C. It was observed that the initial and 10 min gel strength of the drilling fluid show the same trend as the rpm value under R_3_. At 25 °C, the gel strength of the drilling fluid was slightly reduced, but after high-temperature aging, it showed temperature resistance, maintained the stability of the drilling fluid gel system, and thus maintained the viscosity and gel strength of the drilling fluid at high temperatures.

### 2.6. Mechanism of Sodium Acetate Improving the High-Temperature Stability of Bentonite

**Figure 11:**
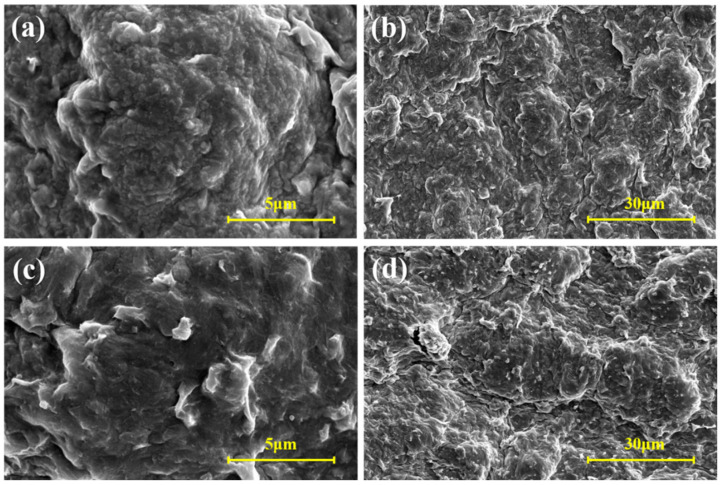
Surface morphology of JGN: (**a**) 0% TRSA—5 μm; (**b**) 0% TRSA—30 μm; (**c**) 2% TRSA—30 μm; (**d**) 2% TRSA—30 μm.

**Figure 12:**
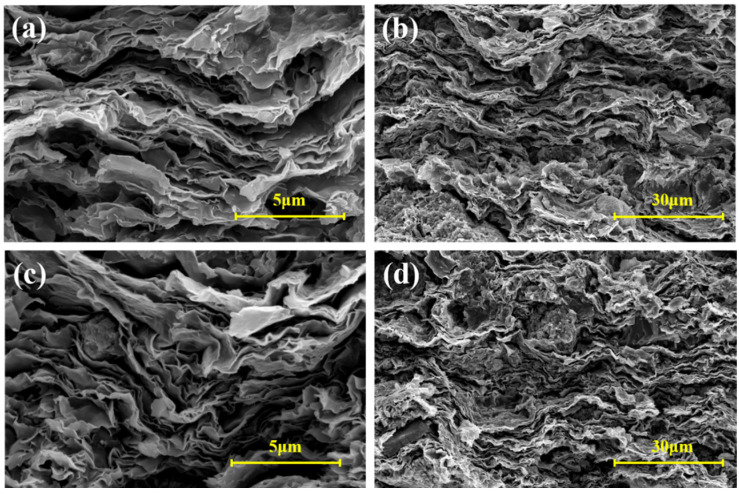
Cross-sectional morphology of JGN: (**a**) 0% TRSA—5 μm; (**b**) 0% TRSA—30 μm; (**c**) 2% TRSA—30 μm; (**d**) 2% TRSA—30 μm.

The micro-morphology of bentonite in drilling fluids with 0% and 2% TRSA additions (based on bentonite JGN) after aging at 180 °C was analyzed by scanning electron microscopy (SEM, Thermo Fisher Corporation, Waltham, MA, USA). Figure 11 show the comparative surface morphologies, while Figure 12 presents the comparative cross-sectional morphologies. It can be clearly observed from the figures that bentonite exhibits a layered structure, and the particles of bentonite after high-temperature aging are mainly present in a face-to-face structure, with a small amount of edge-to-face structure interspersed. Figure 11a,b show the surface morphologies of JGN without TRSA addition at different magnifications, while Figure 11c,d show the surface morphologies of JGN with 2% TRSA addition at different magnifications. A comparison between Figure 11a and Figure 11c reveals that there are numerous dot-like aggregates in Figure 11a, whereas the surface of Figure 11c is relatively smooth. This phenomenon may be attributed to the aggregation of bentonite particles induced by high temperature.

The dominant interaction between sodium acetate and bentonite is hydrogen bonding [46]. When sodium acetate (CH_3_COONa) dissolves in water, the dissociated Na^+^ participates in the ion exchange reaction between the interlayer cations of bentonite. Meanwhile, the CH_3_COO^−^ anions, as species with high hydrogen bond density, generate strong van der Waals forces with the hydroxyl groups (-OH) on the bentonite surface, thereby enhancing the apparent viscosity and shear stress of the drilling fluid.

**Figure 13:**
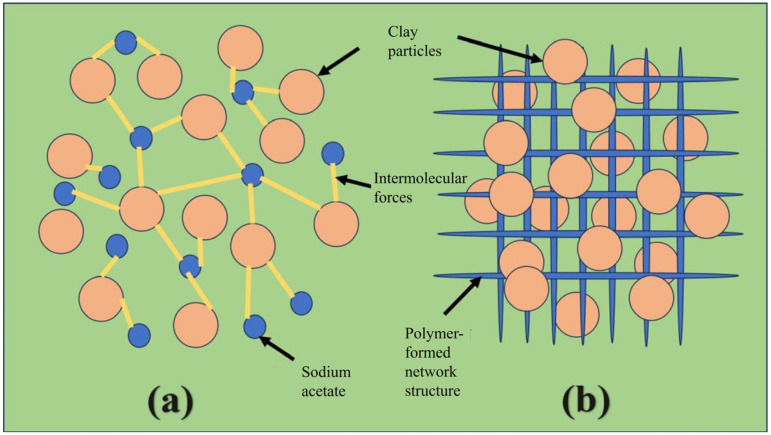
Mechanism simulation diagram: (**a**) Sodium acetate; (**b**) polymer. Note: Orange circles denote clay particles, blue circles denote sodium acetate, yellow lines denote intermolecular forces, and blue grids denote the polymer-formed network structure.

Figure 13 presents a comparison of the action mechanisms between polymers and sodium acetate in clay. Figure 13a illustrates the network structure formed between sodium acetate and bentonite, which is analogous to the spatial structure of ionic crystals. As a monomeric unit, sodium acetate connects with individual clay particles via strong intermolecular forces to construct a three-dimensional network, endowing the drilling fluid with a certain structural strength. Figure 11b depicts the spatial structure formed by macromolecular polymers and bentonite, where the self-assembled three-dimensional polymer network immobilizes clay particles through adsorption and coating effects, and the rigidity derived from the polymer molecular weight contributes to the structural strength of the drilling fluid.

The mechanism by which sodium acetate enhances the performance of bentonite-based drilling fluids differs from that of polymers. Polymers increase the viscosity partially through intermolecular forces and partially through the physical effects provided by their long, rigid molecular chains. In contrast, sodium acetate lacks long molecular chains to adsorb and entangle with bentonite particles. At high shear rates, the intense shear force disrupts the hydrogen bonds formed between molecules, resulting in a relatively low viscosity of the drilling fluid under high shear conditions. By comparison, the rigidity of polymers enables the drilling fluid to maintain high viscosity even under strong shear forces.

At low shear rates, sodium acetate can form a high-density hydrogen bond network, which allows for the rapid reconstruction of the spatial structure, thus improving the gel strength of the drilling fluid [47]. The interaction between sodium acetate and bentonite endows the drilling fluid with a breakable weak-gel structure, which imparts superior shear-thinning behavior to the drilling fluid.

## 3. Conclusions

This study systematically explored the effect of sodium acetate (a non-polymer treatment agent) on the high-temperature rheological properties of calcium-based bentonite water-based drilling fluids, clarifying its dual mechanism as a composite sodium-modifier and rheological modifier and demonstrating the construction of a reversible “weak gel” structure via Na^+^-Ca^2+^ ion exchange between bentonite layers and high-density hydrogen bonding between CH_3_COO^−^ and surface hydroxyl groups of bentonite.

The optimal dosage was confirmed as 2 wt.% TRSA. After aging at 180 °C, the drilling fluid’s low-shear-rate (5.11 s^−1^) viscosity increased from 250 to 1400 mPa·s, gel strength increased from 0.511 to 15.33 Pa, while high-shear-rate (1022 s^−1^) viscosity remained unchanged, effectively balancing cutting suspension and drilling efficiency. Without complex modification, TRSA was applicable to seven bentonite types with different origins and sodium modification states, exhibiting performance equivalent to industrial-grade and analytical-grade products with remarkable economic benefits. The results showed that filtration loss could be maintained through extrusion sodium modification or compounding with sodium polyacrylate (KPAA).

Limitations to this study include unclear long-term stability under ultra-high temperature (>180 °C) and high-salinity synergy, as well as unstandardized critical impurity content and performance consistency of TRSA from different sources. Future research will focus on on-site pilot verification, TRSA impurity purification technology development, and expanded applicability in oil-based/synthetic-based drilling fluids. This study achieves the precise regulation of drilling fluids’ high-temperature rheological properties simply and efficiently, providing an effective approach for the high-value utilization of by-products and new insights for designing high-temperature-resistant drilling fluid systems for deep wells, with both technical innovation and engineering application value.

## 4. Materials and Methods

### 4.1. Experimental Materials

**Figure 14:**
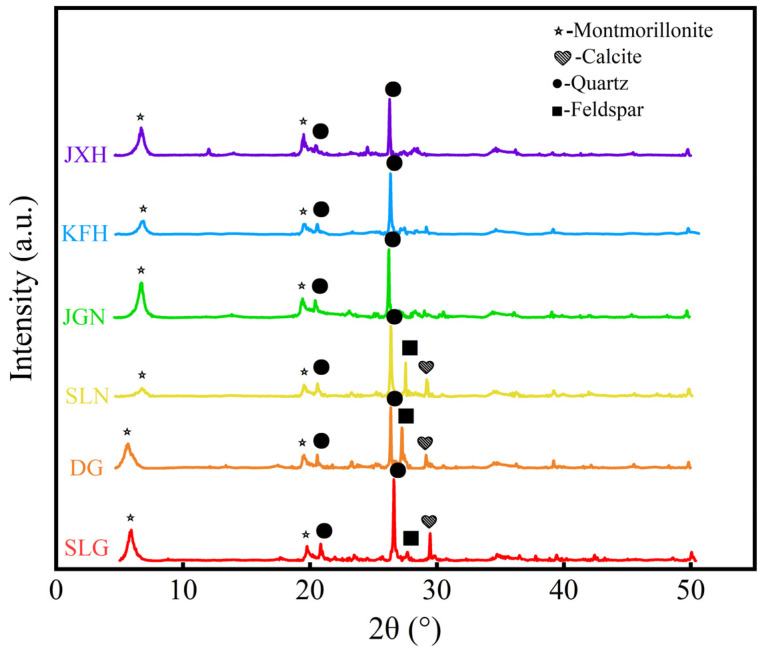
Component analysis of bentonite samples by XRD.

In the experiment, several types of bentonites from different origins in China, denoted as DG, JXH, XH, XHYB, JGN, SLN, and SLG, were selected. Among them, DG and SLG are calcium-based bentonites without sodium modification; JXH, XH, XHYB, JGN, and SLN are calcium-based bentonites that have undergone sodium modification. Table 7 shows the composition analysis of the bentonite samples. The main components of the oxides are Na_2_O, MgO, Al_2_O_3_, SiO_2_, K_2_O, CaO, TiO_2_ and Fe_2_O_3_. The ratio of CaO/Na_2_O in the unmodified bentonite is higher than that in bentonite with sodium modification. The order of silicon–aluminum ratio from large to small is SLG (3.47) > SLN (3.34) > DG (3.13) > JGN (3.01) > KFH (2.98) > JXH (2.43). Figure 14 shows that montmorillonite, quartz, calcite and feldspar are the main components of bentonite. The characteristic peak of montmorillonite is at 5–7°. The characteristic peak of montmorillonite shifted from 5.8° to 7° after sodium modification. The characteristic peaks of quartz are at 21° and 26.69°. The characteristic peak of calcite is at 29.5°. The characteristic peak of feldspar is about 27.7°.

By-product sodium acetate (TRSA), Tianjin Taishen Haotian Chemical Co., Ltd. (Tianjin, China); two types of industrial-grade sodium acetate trihydrate (TSA, SSA), Shijiazhuang Chenxiang Mining Co., Ltd., (Shijiazhuang, China); sodium acetate (A), Beijing Chemical Plant (Beijing, China); anhydrous sodium carbonate, AR, Chemical Reagent Co., Ltd., of Sinopharm Group (Shanghai, China); magnesium oxide (MgO), AR, Chemical Reagent Co., Ltd., of Sinopharm Group (Shanghai, China); carboxymethyl cellulose (CMC), Wandu Petroleum Technology Co., Ltd., (Xianyang, China); hydrolysis of sodium polyacrylonitrile (NaPAN), Santuo Chemical Products Co., Ltd., (Baoding, China); industrial-grade sodium polyacrylate (KPAA), Chaoyang Fanuo Water Purification Materials Co., Ltd., (Zhengzhou, China); 30-million-molecular-weight sodium polyacrylate, AR, Aladdin (Shanghai, China); and 5-million-molecular-weight sodium polyacrylate, AR, Aladdin (Shanghai, China).

### 4.2. Preparation of Drilling Fluid

The drilling fluid performance was evaluated in accordance with Q/SY 17009-2019 [48] as follows: 22.5 g of bentonite sample and the corresponding additives were added to a mixing cup containing 350 mL of water, followed by high-speed stirring (Qingdao Haitongda Special Instruments Factory, Qingdao, China) at 11,000 r/min for 20 min. The stirred drilling fluid was transferred to a curing tank, and its room-temperature performance was measured after 16 h of curing. The 16 h cured drilling fluid was re-stirred for 5 min before being placed into an aging tank, which was subjected to a 16 h aging experiment in a hot-rolling furnace (Qingdao Chuangmeng Instrument Co., Ltd., Qingdao, China) at 150 °C and 180 °C, respectively. After the hot-rolled aging tank was cooled to 25 °C in water, the performance of the aged drilling fluid was determined.

### 4.3. Measurement of Drilling Fluid Viscosity and Shear Force

The viscosity and shear force of drilling fluid were determined by GB/T 16783.1 [49]. Drilling fluid was added to the sample cup, and the drilling fluid was tested using a rotary viscometer, Fann35 (Qingdao Chuangmeng Instrument Co., Ltd., Qingdao, China). After the dial stabilized, the values of R_600_, R_300_, R_200_, R_100_, R_6_, and R_3_ were read in the dial. After the drilling fluid was stationary for 1 min, the maximum deflection value R_3_ (1 min) of the R_3_ gear was recorded; after the drilling fluid was stationary for 10 min, the maximum deflection value R_3_ (10 min) of the R_3_ gear was recorded.

### 4.4. Determination of API Filtration Rate

The API filtration rate of the drilling fluid was determined in accordance with GB/T 16783.1 [49]. The drilling fluid was poured into a filter (Qingdao Tongchun Instrument Co., Ltd., Qingdao, China), and the filtration rate of the drilling fluid was measured at a pressure of 690 KPa.

### 4.5. Extrusion Sodium Modification

Dissolve the sodium agent in 40 mL of water and evenly spray it onto the bentonite, stirring evenly. Use a manual extrusion sodium machine (Beijing Investigation Office, Beijing, China) to extrude the bentonite, repeatedly extrude 7 times, and then age at room temperature for 7 days. After drying and crushing, extruded sodium bentonite is obtained.

### 4.6. Swelling Volume Testing Method

The swelling volume of bentonite was tested by GB/T 20973-2020 [50]. First, a 100 mL stoppered graduated cylinder was filled with 50 mL of water. Then, an analytical balance (Changzhou Lucky Electronic Equipment Co., Ltd., Changzhou, China) was used to weigh 1 g of the bentonite sample, which was added to the plug measuring cylinder; the cylinder was shaken up and down 300 times. Subsequently, 25 mL of hydrochloric acid was added, and additional water was added until the 100 mL mark. It was shaken up and down 100 times and allowed to stand for 24 h. Finally, the scale value (mL/g) of the hydrated bentonite was read and recorded.

### 4.7. Component Analysis Method

XRD analysis was performed using a D8 ADVANCE X-ray diffractometer (Brook Company, Berlin, Germany). The tube current was 40 mA, the tube voltage was 40 kV, and the Cu target wavelength was 1.5406 Å. The scanning angle range is 5–90°.

XRF analysis was performed using the ARLAdvantX IntellipowerTM3600 X-ray fluorescence spectrometer (Thermo Fisher Scientific, Waltham, MA, USA).

### 4.8. Measurement of Filter Cake Permeability

After the 30 min filtration test, the filter cake was carefully removed from the filter paper to avoid structural damage. A digital caliper was used to measure the thickness of the filter cake, taking at least three points and averaging the measurements. According to Darcy’s law, the filter cake permeability K is calculated as:

(2) $$K\;=\;\frac{Q\cdot \mu L}{A\cdot ∆P}$$

Q: filtrate volume flow rate (mL/s); μ: filtrate viscosity (mPa·s); L: filter cake thickness (cm); A: filter cake area (cm^2^); ΔP: pressure differential (psi, typically 100 psi).

## 5. Economic Costs and Environmental Factors

### 5.1. Comparison of Raw Material Costs

The raw material cost of treatment agents directly determines the overall cost of drilling fluid formulations. As shown in Table 8, traditional polymer treatment agents, such as the hydrophobic associative polymer ASML and zwitterionic copolymer DADN, require complex synthesis processes including grafting, cross-linking, and monomer copolymerization, resulting in high production costs ranging from 15,000 to 30,000 RMB per ton. Even conventional inorganic sodium-modifying agents (e.g., analytical-grade anhydrous sodium carbonate) have a market price of approximately 3500–4000 RMB per ton. In contrast, by-product sodium acetate (TRSA) is derived from waste recycling in coal chemical or wet-process phosphoric acid production, with a cost of only 2000–3000 RMB per ton, which is merely 1/5–1/10 of that of polymer treatment agents and 60–80% of that of analytical-grade sodium carbonate. Industrial-grade sodium acetate (TSA/SSA) and analytical-grade sodium acetate (A) also exhibit significant cost advantages over polymers, with price ranges of 3000–5000 RMB per ton.

The optimal dosage of treatment agents further amplifies the economic benefits of sodium acetate. Experimental results indicate that the optimal addition amount of TRSA as a composite sodium-modifying agent and rheological modifier is only 2 wt.%, which is significantly lower than the dosage of traditional polymer treatment agents (usually 3–5%). Taking a single well with a drilling fluid demand of 1000 tons as an example, the cost of using TRSA is only 40,000–60,000 RMB, while the cost of using polymer treatment agents ranges from 450,000 to 1,500,000 RMB, and the cost of using pure sodium carbonate is 140,000–160,000 RMB. Additionally, TRSA integrates dual functions of sodium modification and rheological regulation, eliminating the need for additional suspending agents or rheological modifiers (which are required for traditional sodium carbonate-based sodium modification). This reduces the types of additives by 30%, further lowering the comprehensive formulation cost.

### 5.2. Environmental Impact Assessment

In line with global low-carbon development trends and environmental protection requirements for drilling operations, the environmental friendliness of treatment agents—including biodegradability, waste emissions, and resource utilization efficiency—has become a crucial evaluation index. As an inorganic treatment agent based on industrial by-products, sodium acetate demonstrates significant environmental advantages compared to synthetic polymer treatment agents.

Drilling waste fluids containing polymer treatment agents require complex treatment processes (such as advanced oxidation and flocculation precipitation) to meet discharge standards, with a treatment cost of 80–120 RMB per ton. In contrast, waste fluids containing sodium acetate have a simple composition, and their pH value can be adjusted to neutral through natural dilution or simple acid–base neutralization, with a treatment cost of only 10–20 RMB per ton. For a single well generating 500 tons of waste fluid, the use of TRSA can save 35,000–55,000 RMB in waste fluid treatment costs, further enhancing its comprehensive economic and environmental benefits.

## Figures and Tables

**Table 1:** Different types and contents of sodium agents.

Name	Type and Dosage of Sodium Carbonate
4SC	4% Na_2_CO_3_
2SC2TRSA	2% Na_2_CO_3_ + 2 %TRSA (By-product grade sodium acetate)
2SC2TSA	2% Na_2_CO_3_ + 2 %TSA (Industrial grade sodium acetate)
2SC2SSA	2% Na_2_CO_3_ + 2 %SSA (Industrial grade sodium acetate)
2SC2A	2% Na_2_CO_3_ + 2% A (Analytical pure anhydrous sodium acetate)

**Table 2:** Dynamic ratio and filtration loss of different sodium agents at room temperature and high temperature.

Sodium Agents	RYP (25 °C/150 °C/180 °C)	FL (25 °C/150 °C/180 °C)
4SC	2.48/1.05/0.44	16.4/21.6/21.8
2SC2TRSA	2.64/2.56/1.64	19.8/26.6/30
2SC2TSA	2.90/25.4/1.94	19/25.4/26.5
2SC2SSA	2.90/1.95/0.82	17.8/24/24.2
2SC2A	2.56//1.28	17.6//27.2

**Table 3:** The dynamic speed ratio and filtration loss of SLG using different sodium agents at room temperature and high temperature after extrusion sodium modification.

Sodium Agents	RYP (25 °C/150 °C/180 °C)	FL (25 °C/150 °C/180 °C)
4SC	0.43/0.26/0.30	16.8/21/17.8
2SC2TRSA	0.34/0.41/0.42	26/30.4/25.2
J4SC	0.73/0.31/0.31	11.2/19.2/19
J2SC2TRSA	0.61/0.56/0.66	16.2/22.6/24.4
J4SC2TRSA	1.02/0.37/0.16	15.2/20.4/20.4

**Table 4:** The dynamic speed ratio and filtration loss of JXH using sodium acetate as a rheological regulator at room and high temperatures.

Regulator	RYP (25 °C/150 °C/180 °C)	FL (25 °C/150 °C/180 °C)
None	1.07/1.48/0.89	14.2/17/19.6
2% TRSA	2.64/1.66/0.97	14.4/18.4/18.2
2% TSA	1.72/1.83/1.19	17.2/19/18
2% A	2.11/1.65/0.93	16.8/19/18.4

**Table 5:** Effects of sodium acetate and sodium polyacrylate on the rheological properties of JGN from top to bottom are as follows: ① No addition; ② 2% TRSA; ③ 0.1% KPAA (industrial-grade sodium polyacrylate); ④ 0.1% KPAA + 2% TRSA.

	T	Viscometer Readings						Rheological Properties					FL (mL)	K (mD)
		R_600_	R_300_	R_200_	R_100_	R_6_	R_3_	AV_600_	AV_6_	PV	YP	RYP		
	25	25	16	12.5	8.5	3	3	12.5	150	9	3.58	0.40	12.8	11.8
①	150	55.5	35.5	26	16	4	3	27.75	200	20	7.92	0.40	14.8	17.8
	180	73	50	39	26.5	7.5	6	36.5	375	23	13.80	0.60	12.2	12.0
	25	26	19	16	12	7.5	7.5	13	375	7	6.13	0.88	12	12.8
②	150	43.5	29	22.5	15	6.5	6	21.75	325	14.5	7.41	0.51	14.4	15.06
	180	59	41	33	23	9	8	29.5	450	18	11.75	0.65	16	20.0
	25	82	60	57	39.5	22	21.5	41	1100	22	19.42	0.88	11.6	12.0
③	150	112	85	73	58	38	37	56	1900	27	29.64	1.10	12.2	12.3
	180	72	47	36	23	4	2.5	36	200	25	11.24	0.45	13	16.1
	25	54	39	31.5	24	15	15	27	750	15	12.26	0.82	13.2	13.5
④	150	75	52	41	30	16	16	37.5	800	23	14.82	0.64	15.8	18.6
	180	75	53	43	32	16	14	37.5	800	22	15.84	0.72	15.6	18.7

**Table 6:** Fitting results of the Herschel–Bulkley model for JGN bentonite drilling fluid under different additives and temperatures from top to bottom are as follows: ① no addition; ② 2% TRSA; ③ 0.1% KPAA (industrial-grade sodium polyacrylate); ④ 0.1% KPAA + 2% TRSA.

	T	τy (Pa)	K (Pa·s^n^)	n	R^2^		τy (Pa)	K (Pa·s^n^)	n	R^2^
①	25	2.8	0.015	0.75	0.998	③	15.5	0.025	0.68	0.997
	150	5.5	0.032	0.70	0.997		25.0	0.035	0.62	0.996
	180	8.9	0.045	0.65	0.996		7.5	0.038	0.69	0.995
②	25	5.2	0.008	0.85	0.999	④	10.0	0.015	0.73	0.998
	150	5.8	0.020	0.78	0.998		12.2	0.025	0.70	0.998
	180	8.1	0.028	0.72	0.997		13.0	0.024	0.71	0.998

**Table 7:** Composition of bentonite samples (wt%).

Bentonite Sample	Na_2_O	MgO	Al_2_O_3_	SiO_2_	K_2_O	CaO	TiO_2_	Fe_2_O_3_
SLG	0.384%	2.687%	18.509%	64.172%	2.723%	5.340%	0.695%	4.792%
DG	0.835%	2.729%	19.687%	61.689%	2.563%	3.966%	0.634%	7.098%
SLN	2.572%	2.865%	18.684%	62.466%	2.130%	4.606%	0.739%	5.025%
JGN	3.138%	2.959%	20.511%	62.572%	2.092%	3.548%	0.390%	3.588%
KFH	3.103%	2.679%	20.508%	61.119%	2.170%	3.751%	0.584%	4.478%
JXH	3.915%	3.681%	24.700%	60.105%	0.900%	1.865%	0.557%	2.872%

**Table 8:** Cost comparison of different drilling fluid treatment agents.

Treatment Agent Type	Raw Material Cost (yuan/ton)	Optimal Dosage (wt.%)	Comprehensive Cost for 1000-ton Drilling Fluid (RMB)
TRSA	2000–3000	2	40,000–60,000
TSA/SSA	3000–5000	2	60,000–100,000
A	5000–8000	2	100,000–160,000
AR	3500–4000	4	140,000–160,000
ASML	20,000–30,000	3–5	600,000–1,500,000
DADN	15,000–25,000	3–4	450,000–1,000,000

## Data Availability

The original contributions presented in this study are included in the article. Further inquiries can be directed to the corresponding authors.

## Glossary

TRSA	By-product grade sodium acetate	TSA	Industrial-grade sodium acetate trihydrate
RYP	Dynamic speed ratio	API FL	API filtration loss
SSA	Industrial-grade sodium acetate trihydrate	AV600	Apparent viscosity at 600 rpm (mPa·s)
A	Analytical-grade pure anhydrous sodium acetate	AV6	Apparent viscosity at 6 rpm (mPa·s)
Shear rate	Rate of shear applied to drilling fluid (s^−1^)	AV3	Apparent viscosity at 3 rpm (mPa·s)
PV	Plastic viscosity (mPa·s)	YP	Yield point (Pa)
Rpm	Revolutions per minute (viscometer dial reading unit)	φ_3_, φ_6_, φ_100_, φ_200_, φ_300_, φ_600_	Viscometer dial readings at 3/6/100/200/300/600 rpm
τ_0_ (Initial gel strength)	Initial gel strength (10 s) (Pa)	τ_10_ (10 min gel strength)	10 min gel strength (Pa)
KPAA	Industrial-grade sodium polyacrylate	Shear stress	Stress generated by shear force in drilling fluid (Pa)
NaPAN	Sodium polyacrylonitrile hydrolysate	Swelling volume	Hydration swelling volume of bentonite (mL/g)
SA	Sodium acetate	DG	Calcium-based bentonite (Dagang Oilfield)
JXH	Sodium-modified calcium-based bentonite (Hebei Xuanhua Yanbei)	XH	Sodium-modified calcium-based bentonite (Hebei Xuanhua)
JGN	Sodium-modified calcium-based bentonite (Liaoning Chaoyang Kazuo)	SJ	Calcium-based bentonite (Xinjiang Hami)
SLN	Sodium-modified calcium-based bentonite (Shandong Shengli Oilfield)	SLG	Calcium-based bentonite (Shandong Shengli Oilfield)
KFH	Sodium-modified calcium-based bentonite (Liaoning Chaoyang Kazuo)	4SC	4% Na_2_CO_3_
2SC2TRSA	2% Na_2_CO_3_ + 2% TRSA	2SC2TSA	2% Na_2_CO_3_ + 2% TSA
2SC2SSA	2% Na_2_CO_3_ + 2% SSA	2SC2A	2% Na_2_CO_3_ + 2% A
J4SC	4% Na_2_CO_3_ treated by extrusion sodium modification	J2SCSTRSA	2SC2TRSA treated by extrusion sodium modification
J4SC2TRSA	4% Na_2_CO_3_ + 2% TRSA treated by extrusion sodium modification	Å	Angstrom (wavelength unit)
CMC	Carboxymethyl cellulose	HP/HT	High pressure/high temperature
